# Systematic review and narrative synthesis of suicide prevention in high-schools and universities: a research agenda for evidence-based practice

**DOI:** 10.1186/s12889-021-11124-w

**Published:** 2021-06-10

**Authors:** Elsie Breet, Matsie Matooane, Mark Tomlinson, Jason Bantjes

**Affiliations:** 1 Institute for Life Course Health Research, Department of Global Health, Stellenbosch University, Cape Town, South Africa; 2 Department of Psychology, Stellenbosch University, Stellenbosch, South Africa; 3 School of Nursing and Midwifery, Queens University, Belfast, UK

**Keywords:** Suicide prevention, University, High-school, Campus-based, Prevention interventions, Students

## Abstract

**Background:**

Youth suicide prevention in high-schools and universities is a public health priority. Our aim was to propose a research agenda to advance evidence-based suicide prevention in high-schools and universities by synthesizing and critically reviewing the research focus and methodologies used in existing intervention studies.

**Methods:**

Fourteen databases were systematically searched to identify studies which evaluate suicide prevention interventions delivered on high-school or university campuses, with before and after measures. Data from included studies (*n* = 43) were extracted to identify what, where, how and for whom interventions have been tested. Narrative synthesis was used to critically evaluate research focus and methodology. Study quality was assessed.

**Results:**

Research has focused primarily on selective interventions, with less attention on indicated and universal interventions. Most evidence comes from North America and high-income countries. The target of interventions has been: non-fatal suicidal behaviour; confidence and ability of staff/students to intervene in a suicidal crisis; suicide-related knowledge and attitudes; and suicide-related stigma. No studies included suicide deaths as an outcome, evaluated eco-systemic interventions, explored how context influences implementation, used multisite study designs, or focused explicitly on LGBTQ+ youth. Two studies evaluated digital interventions. Quality of the majority of studies was compromised by lack of methodological rigour, small samples, and moderate/high risk of bias. Interventions often assume the existence of an external well-functioning referral pathway, which may not be true in low-resource settings.

**Conclusion:**

To advance evidence-based suicide prevention in educational settings we need to: conduct more high-quality clinical and pragmatic trials; promote research in low- and middle-income countries; test targeted interventions for vulnerable populations (like LGBTQ+ youth), evaluate interventions where death by suicide is the primary outcome; include translational studies and use implementation science to promote intervention uptake; evaluate the potential use of digital and eco-systemic interventions; and conduct multisite studies in diverse cultural settings.

**Supplementary Information:**

The online version contains supplementary material available at 10.1186/s12889-021-11124-w.

## Background

Suicide is the second leading cause of death among young people aged 15 to 29 years old [1] with the first onset of suicidal behaviour typically occuring in late adolescence [2]. Studies have consistently drawn attention to the marked rates of non-fatal suicidal behaviour among high-school and university students [3, 4], with a recent systematic review reporting pooled 12-month prevalence estimates among college students for suicidal ideation, plan, and attempt of 10.6, 3.0 and 1.2%, respectively [5]. A study of 146,460 high-school pupils (aged 12 to 18) in 40 low- and middle-income countries (LMICs) reported a pooled mean 12-month prevalence rate for suicide attempt of 17.2% [6]. Reducing suicidal behaviour among adolescents and young adults is an important public health priority, which requires an understanding of risk and protective factors as well as awareness of the evidence-base for effective interventions [7]. Elevated rates of suicidal behaviour among high schoolers and university students are often attributed to the psychosocial stressors that typically accompany this developmental period, including difficulty adapting to increased academic workload, substance use, bullying, inadequate skills to resolve inter-personal conflicts, and stigma about accessing mental healthcare [8, 9]. Furthermore, suicidal young people are often reluctant to seek help, are less likely to access formal treatment, and do not attend treatment arranged for them [8]. A national survey of college counselling centre directors in Canada and the United States reported that 80% of students who died by suicide on campus had never utilised university counselling services [10]. Despite the availability of campus-based mental health services, many high-school and university students do not access these services because of insufficient knowledge of where to go [11] and lack of time to attend formal treatment [9], which further increases their risk of suicide.

High-school and university campuses are a potential site for targeted suicide prevention, as this environment provides easy and on-going access to a clearly delineated vulnerable population, making it possible to adopt an evidence-based public health approach to youth suicide prevention [12]. Furthermore, adolescents and young adults spend significant amounts of time on campus, which provides opportunities for targeted, cost-effective and sustained interventions [9]. However it is not always clear which interventions are most effective and which may have adverse effects, making it difficult to plan and implement evidence-based public health approaches to campus-based suicide prevention [13, 14]. Identifying an evidence base for campus suicide prevention is important for guiding clinical practice, informing policy, allocating resources and focusing future research [15–17].

Within a public health framework, campus-based suicide prevention strategies can be grouped into universal, selective, and indicated interventions [18–20]. Universal interventions are population level strategies aimed at reaching the whole student body without regard for individual risk factors [21]. Universal prevention programs are designed to influence all students and hence reduce suicide by removing barriers to care and promoting access to mental health services, enhancing knowledge of how to help suicidal individuals, and strengthening protective processes like social support and coping skills [22]. Universal prevention strategies include interventions such as: psychoeducation to increase awareness about suicide, providing information about campus-based crises and mental health services, reducing stigma, and encouraging help seeking [22]. Selective prevention strategies are targeted at subgroups which are known to be at elevated risk of suicide, such as students who are depressed or are the victims of bullying [23]. Examples of selective interventions include screening programs, gatekeeper training for “frontline” adult caregivers (such as counsellors), training peer helpers who can provide support to distressed students, support and skill building groups for at-risk students, enhancing access to crisis and treatment services, and targeted outreach to high-risk groups to provide information about available services [19, 24, 25]. Gatekeeper training is an example of a widely used selective suicide prevention strategy which entails training peers, academic staff, resident assistants, or counsellors to recognise at risk students and encourage them to access appropriate help [26]. Indicated suicide prevention strategies are focused on students who are already experiencing warning signs, reporting suicidal thoughts, and/or who have made a suicide attempt. Indicated interventions are narrowly focused on high-risk individuals in order to engage them in treatment and hence reduce risk of suicide and/or increase protective factors [24]. Indicated interventions can be delivered to individuals or groups, usually by a trained mental health professional [27].

The World Health Organisation recommends that suicide prevention strategies should include universal, selective, and indicated interventions in order to be comprehensive [19]. Isolated interventions which are not sustained and are not part of an integrated multi-level prevention strategy have a very low probability of yielding significant reductions in suicide at a population level [28, 29]. To be effective, campus-based suicide prevention programmes will need to have clearly articulated targets that integrate evidence-based universal, selective, and indicated prevention [30]. Furthermore, they should include evidence-based clinical care for suicidal students alongside population level psychosocial and psychoeducational interventions [31].

Systematic reviews of campus-based interventions assist schools and universities to develop evidence-based integrated, multifaceted, suicide prevention strategies. To-date, 5 systematic reviews have already been done in this area, two of which focused exclusively on high school-based suicide prevention programs [32, 33], two focused exclusively on university-based programmes [15, 26], and one systematic review integrated evidence from high-schools and universities [34]. It makes sense to consider evidence from high-schools and universities together, given that interventions effective with adolescent high-schoolers may also be appropriate and effective for college-aged students, and vice versa. It also seems likely that strategies developed within one of these settings could easily be modified to make them appropriate for the other setting, especially given that there is much overlap in the risk factors for suicidal behaviour among adolescents and young adults [35]. The existing reviews are helpful in synthesizing outcome data from intervention studies, but some are limited by the use of relatively narrow search strategies; for example, Katz et al. only search two data bases [32] and Harrod et al. only reviewed primary prevention programmes [15]. With the exception of Harrod et al’s review of primary interventions in post-secondary educational environments [15], the existing reviews have also neglected to assess the quality of studies and the risk of bias. Nonetheless, the existing reviews provide a detailed presentation of statistical outcome measures [36], and have enabled a meta-analysis of the effectiveness of interventions that have been tested [34]. Our aim in this study was to build on the work of these existing systematic reviews by conducting a narrative synthesis of the available literature to critically review the research methods and research focus of existing studies. We wanted to consider what kinds of strategies have been tested, where, how and for whom, in order to propose a research agenda to advance evidence-based practice in this field.

## Methods

### Study aim and design

The aims of this study are to: (1) critically review the research methods and research focus in the existing evidence-base for high-school and university suicide prevention programmes; and (2) propose a research agenda to advance the practice of suicide prevention in schools and universities. To achieve these aims we first conducted a systematic review of campus-based suicide prevention strategies using an expanded research strategy (i.e. a wider array if search terms and databases than was utilised in previous systematic reviews). Second, we conduct a narrative synthesis focused on answering the question “What kinds of strategies have been tested, where, how and for whom?”. Lastly, we identified gaps in the current research and proposed a research agenda that could close these gaps.

We made use of narrative synthesis because this approach to literature reviews explicitly allows for the presentation of statistical outcome data alongside a textual description and discussion of the study findings [37], providing opportunities to answer a wider range of research questions, beyond only those related to intervention effects [37]. Narrative synthesis is particularly useful in reviews such as this one where the experimental and quasi-experimental studies that have been included are not sufficiently similar to permit a meta-analysis [38].

### Study procedures and setting

For the systematic review component of this study we followed the Preferred Reporting Items for Systematic Reviews and Meta-Analyses (PRISMA) guidelines [39].

#### Search strategy and selection criteria

We searched PubMed/MEDLINE, Cochrane library trials, CINAHL Plus (EBSCO*host*), DARE (Database of Abstracts of Reviews of Effectiveness), Africa-Wide Information (EBSCO*host*), IMSEAR; Korea (Med); EurasiaHealth; SciELO; The Latin American Social Medicine database; East View Information Services; Arctic Health; Medindia.net; and African Journals Online for all studies published in English from the inception of the database until 5 August 2019. We include regional databases in our searches, since global databases do not always include less-prominent but nonetheless respected regional journals. We searched databases from their inception with the aim of trying to identify all interventions that had been tested and to track the evolution of approaches to this public health issue over time. A comprehensive search strategy was developed for PubMed which was adapted for every other database. Exploded MeSH terms and key words relevant to suicide-related behaviour, intervention type, university or school, and trial type were combined using standard Boolean operators (see Supplementary Material, Table S1). We also hand-searched the reference lists of previous reviews to identify additional studies that might meet our inclusion criteria.

Studies were eligible for inclusion if they: (1) were peer-reviewed publications; (2) reported an intervention study with before-and-after outcome measures; (3) targeted as primary outcomes, any form of suicidal behaviour (including suicidal ideation, plan, attempt or suicide), suicide-related knowledge/attitudes, skills to intervene in a suicidal crisis, suicide-related stigma, of help-seeking behaviour; (4) targeted high-school or university students or staff working in these environments, and entailed interventions that were delivered on campus (i.e., campus-based); and (5) were published in English.

Two researchers working independently screened all identified articles by title and abstract to eliminate papers which clearly did not meet the inclusion criteria. Subsequently, the full texts of potentially relevant studies were independently screened by two researchers for inclusion in the review. The results from the independent screeners were then sent to a third researcher, who compared the results and compiled a list of included studies. Discrepancies between the results from both researchers were discussed with the third researcher until agreement was reached.

#### Data extraction and management

Data were independently extracted by two researchers and subsequently checked by a third researcher. The following data were extracted and captured on excel spreadsheets: author and year of publication; site of intervention (high-school versus university campus), intervention target population; gender composition of the study sample; number of participants randomised to intervention; intervention period in weeks; duration of each contact session in minutes; number of sessions; study region and economic classification of the country where the study was conducted; details of the intervention; study design; target of the intervention; main findings; and effect size. We contacted the authors of any studies that did not report the necessary data to request this information.

#### Study quality

Study quality was assessed using the Cochrane risk of bias tool for randomised controlled trials (RCTs) [40] and the ROBINS-I tool for assessing risk of bias in non-randomized studies of interventions [41].

#### Description of materials

Not applicable.

### Data analysis

Narrative synthesis was used to summarise the results within a public health framework. Although narrative synthesis does not consist of a set of definitive rules for data analysis, this approach has four main elements, which we have followed closely in this study: 1) locating the intervention within a framework that relates to how, why and for whom the intervention works; 2) developing a preliminary synthesis of included study findings such as setting up tables that optimise the researchers ability to identify patterns across the studies; 3) exploring relationships within the data and considering all potential factors that might explain the direction or size of the effect of the intervention across included studies; and 4) assessing the robustness of the data by commenting on the strengths and weaknesses of the data and highlighting study specific factors or barriers to implementation that might explain discrepancies across study findings [37]. In accordance with these central elements of narrative synthesis, we have presented our findings within a public health framework thus stratifying interventions according to universal campus-wide preventions, selective gatekeeper related preventions, and indicated interventions for high-risk students. The characteristics of the interventions are synthesised in a way that allows a summary and critical discussion of the research focus that has hitherto dominated the work in this field. We reported effect size statistics as they were reported in the respective studies. Where studies did not report effects sizes, we calculated Cohen’s *d* effect size statistics provided that the necessary statistics were reported. To avoid bias related to over/under-counting, the unit of analysis was the intervention rather than the publication. Where a study compared interventions, we treated the intervention as the unit of analysis and not the study.

## Results

**Fig. 1:**
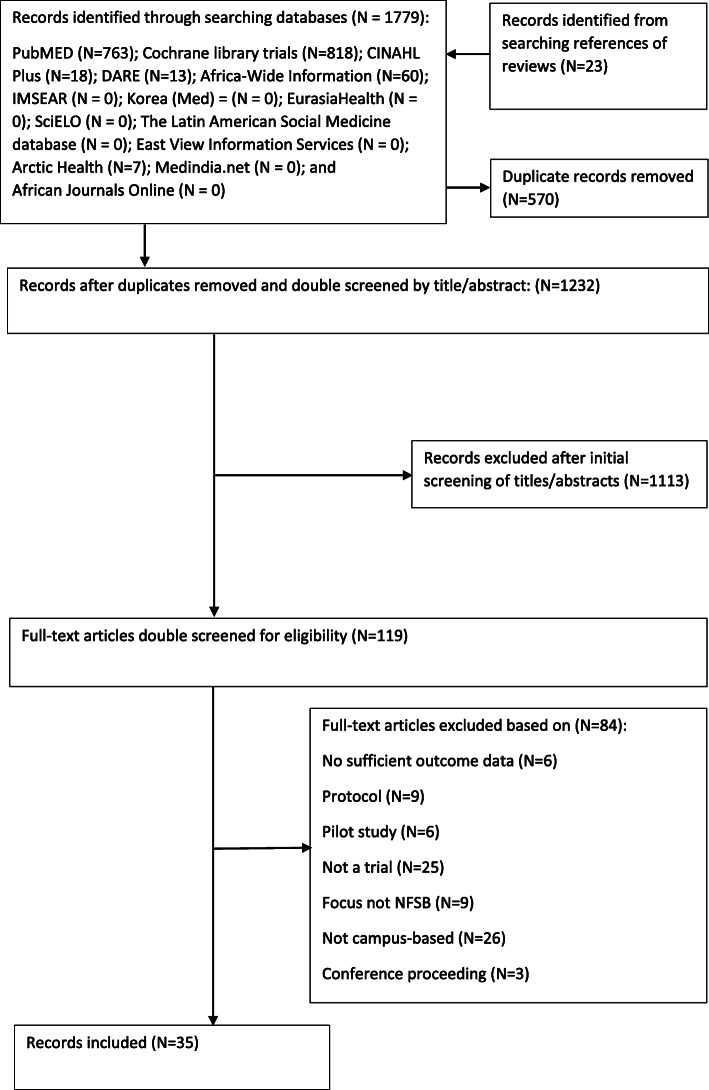
Prisma flowchart of Study Selection

As shown in Fig. 1, the initial searches of electronic databases yielded 1779 articles. We identified a further 23 records from the reference lists of related systematic reviews. A total of 570 duplicate records were removed. Titles and abstracts were screened, and 1113 articles were excluded, leaving 119 studies for full text screening, of which 35 studies met the inclusion criteria. The earliest study identified was published in 1995 with a steady increase in the number of studies observed in the last 10 years (see Supplementary Figure S1). A total of 43 interventions were identified across the included studies.

### Overview of the interventions

The characteristics of the interventions and research methods used to test each intervention, are provided as supplementary material (see Supplementary Table S2). The majority (*n* = 24) of interventions were conducted on high-school campuses, while 19 interventions were tested on university campuses. Studies were primarily conducted in North America (*n* = 26), while smaller proportions were based in the East Asia and Pacific region (*n* = 7), Europe and Central Asia (*n* = 1), and Latin America and the Caribbean (*n* = 1). None of the studies we identified were conducted in Middle East and North Africa, South Asia, and Sub-Saharan Africa. The vast majority of studies were conducted in high-income countries (*n* = 33), with only 1 in an upper-middle-income country, 2 in lower-middle-income countries, and none in low income countries. Of the 43 interventions we identified, 36 showed some significant positive impact, 7 had no impact, and none of the interventions caused an increase in suicide related outcomes. A total of 24,270 participants (61.0% female, 38.0% male, 0.4% transgender, 1.0% non-binary) were included in the intervention groups across the 42 interventions studies that reported sample sizes (mean = 592, S.D. = 1611.1, Range = 12–9666), with 30 of the studies having less than 200 participants. Most interventions were conducted in eight sessions or less (*n* = 26 of 37, Rane = 1–180), and 15 interventions were delivered in a once-off single session. Among those studies that reported the intervention duration in minutes, approximately half (*n* = 17 of 32) of the interventions were administered in 60 min or less with the longest intervention lasting 960 min. Of the 32 interventions that reported duration in weeks, the interventions ranged from less than a week (*n* = 10) to 36 weeks with a mean of 9 weeks. Most of the 35 included studies employed a RCT (*n* = 17) research design, with a smaller proportion being open trials (*n* = 7), controlled before and after studies (*n* = 2), three group repeated measures design (*n* = 1), experimental three-group design (*n* = 1), a single-arm follow-up study (*n* = 1), between-subjects study (*n* = 1), Solomon four-group study design (*n* = 1), and quasi-experimental study (*n* = 4).

### Quality assessment

The quality assessment for RCTs evaluated risk of bias due to the randomization process, intended interventions, measurement of outcomes, and selected reporting of results (see Supplementary Table S3). Among the RCTs (*n* = 17), 10 were assessed as having some or high-risk of bias. The quality assessment for non-RCTs evaluated risk of bias due to confounding factors, recruitment procedures, classification of intervention, deviations from intended intervention, missing data, measurement of outcomes, and selected reporting of results (see Supplementary Tables S4). Among the non-RCTs (*n* = 19), 14 showed moderate to serious risk of bias.

### Universal campus-wide interventions

Fourteen interventions employed universal suicide prevention strategies targeted at the entire student population. These interventions focused on reducing non-fatal suicidal behaviour (NFSB) (*n* = 11), changing knowledge, attitudes and/or stigma about suicide (*n* = 5), and increasing students’ help seeking behaviours (*n* = 3). The characteristics and outcomes of these universal interventions are shown in Table 1 and discussed in more detail below.

**Table 1:** Main outcomes of studies on universal campus-wide interventions

Authors (year of publication)	Details of the intervention	Study design	Target of the intervention	Main findings	Effect Size
Aseltine et al., 2004 [42]	Signs of Suicide (SOS) prevention program	RCT (wait-list control group with follow up at 3 months postintervention)	Reduce suicidal ideation	No significant reduction in suicidal ideation.	Small (*d* = 0.15)
			Reduce suicide attempts	Significant reduction in suicide attempts.	Small (*d* = 0.26)^a^
			Change knowledge	Significant increase in knowledge.	Small (*d* = 0.38)^a^
			Change attitudes	Significant increase in adaptive attitudes toward suicide.	Small (*d* = 0.40)^a^
			Increase in help-seeking behaviour	No significant increase in help seeking in the form of treatment. No significant increase in help seeking from a friend. No significant increase in help seeking from an adult.	Small (*d* = 0.12) Small (*d* = 0.04) Small (*d* = 0.13)
Aseltine et al., 2007 [43]	Signs of Suicide (SOS) prevention program	RCT (wait-list control group with follow up at 3 months postintervention)	Reduce suicidal ideation	No significant reduction in suicidal ideation.	Small (*d* = 0.10)
			Reduce suicide attempts	Significant reduction in suicide attempt.	Small (*d* = 0.26)
			Change knowledge	Significant increase in knowledge about suicide.	Small (*d* = 0.32)
			Change attitudes	Significant increase in adaptive attitudes toward suicide.	Small (*d* = 0.10)
			Increase in help-seeking behaviour	No significant increase in help seeking in the form of treatment. No significant increase in help seeking from a friend. No significant increase in help seeking from an adult.	Small (*d* = 0.02) Small (*d* = 0.02) Small (*d* = 0.08)
Britton et al., 2014 [44]	Classroom-based, teacher implemented, mindfulness meditation intervention	RCT (active control condition with postintervention follow up only)	Reduce suicidal ideation Reduce suicidal behaviour	Significant reduction in suicidal ideation and self-harm.	Large (*d =* 1.13)
Kalafat & Elias, 1994 [45]	Suicide awareness curriculum	Solomon four-groups design (physical education content control group with postintervention follow up only)	Change knowledge	Significant increase in suicide knowledge.	Large (*d* = 0.91)
			Change attitudes	Significant change in positive attitudes towards suicide and helping others.	Small (*d* = 0.17)
Nasution et al., 2019 [46]	Combination of training for mental health nurses (TKN), CBT and a peer leadership (PL) program	Quasiexperimental pre-post test design (care as usual control group with postintervention only follow up)	Reduce suicidal ideation	Significant reduction in suicidal ideation.	Insufficient statistics
Randell et al., 2001 [47]	C-CARE - Counselors CARE	Experimental three-group design (care as usual control group with follow up at 4-weeks and 10-weeks)	Reduce suicide risk: unspecified	Significant reduction in suicide risks behaviours (thoughts, threats, and attempts) in intervention and control group.	Large (*d* = 0.90)
Randell et al., 2001 [47]	CAST - C-CARE plus a 12-session Coping and Support Training	Experimental three-group design (care as usual control with follow up at 4-weeks and 10-weeks)	Reduce suicide risk: unspecified	Significant reduction in suicide risks behaviours (thoughts, threats, and attempts) in intervention and control group.	Large (*d* = 0.90)
Rogers et al., 2018 [48]	Psychoeducation and Interpersonal exposure	RCT (control group browsed the National Diabetes Education website with a postintervention and 1-month follow up)	Reduce stigma	Significant reduction in stigma of suicide.	Small (*d* = 0.46)
				Significant greater reduction in suicide-related stigma among those with prior exposure to suicide at post-intervention.	Small (*d* = 0.28)
				Significant greater reduction in suicide-related stigma among those with prior exposure to suicide at one-month follow-up.	Small (*d* = 0.40)
Schilling et al., 2016 [49]	Signs of Suicide (SOS) prevention program	RCT (wait-list control group with a 12-weeks post-baseline follow up)	Reduce suicidal ideation	No significant reduction in suicidal ideation.	Small (*d* = 0.01)
			Reduce suicide plan	Significant reduction in suicide plan.	Large (*d* = 1.05)
			Reduce suicide attempts	Significant reduction in suicide attempt.	Large (*d* = 0.72)
			Change knowledge	Significant increase in knowledge.	Small (*d* = 0.28)
			Change attitudes	Significant change in adaptive attitudes about suicide.	Small (*d* = 0.05)
Wasserman et al., 2015 [50]	Question, Persuade, Refer (QPR)	Cluster-RCT (control group exposed to six educational posters displayed in their classrooms with a 3-month and 12-month follow up)	Reduce suicidal behaviour	No significant reduction in suicidal behaviour at 3-month follow up.	Small (*d* = −0.26)
				No significant reduction in suicidal behaviour at 12-month follow-up.	Small (*d* = −0.20)
Wasserman et al., 2015 [50]	ProfScreen	Cluster-RCT (control group exposed to six educational posters displayed in their classrooms with a 3-month and 12-month follow up)	Reduce suicide attempts	No significant reduction in likelihood of suicide attempt at 3-month follow-up.	Small (*d* = − 0.14)
				Significant reduction in likelihood of suicide attempt at 12-month follow-up.	Large (*d* = −0.44)
Wasserman et al., 2015 [50]	Youth Aware of Mental Health Programme (YAM)	Cluster-RCT (control group exposed to six educational posters displayed in their classrooms with a 3-month and 12-month follow up)	Reduce suicide attempts	No significant reduction in the likelihood of suicide attempts at 3-month follow-up.	Small (*d* = − 0.14)
				No significant reduction in the likelihood of suicide attempts at 12-month follow-up.	Small (*d* = −0.44)
Wulandari et al., 2019 [51]	Peer leadership training (team formation and building, adolescent related projects, and team activities)	Quasi-experimental pretest–posttest design (no control group with postintervention follow up)	Reduce suicidal ideation	Significant reduction in suicidal ideation.	Insufficient statistics
Wyman et al., 2010 [52]	Source of Strengths prevention program	RCT (wait-list control group with postintervention and 1 year follow up)	Increase help-seeking behaviour	Significant increase in positive expectation that adults at school would help suicidal students.	Medium (*d* = 0.75)^a^
				Significant increase in norms for help-seeking from adults at school.	Medium (*d* = 0.62)^a^
				No significant increase in connecting distressed peers to adults.	Small (*d* = 0.21)^a^

^a^Effect size calculated by the authors and reported as it is reported in the original study

#### Reducing non-fatal suicidal behaviour

Of the 11 interventions that targeted NFSB in the general student population, only three significantly reduced the prevalence of suicidal ideation [44, 46, 51], three significantly reduced prevalence of suicide plans [44, 46, 49], and four significantly reduced prevalence of suicide attempts [42–44, 49]. One study reported a significant reduction in “suicide risk”, which was defined as composite measure of suicide related thoughts, threats, and attempts [47]. As seen in Table 1, the interventions effective for reducing NFSB included the *Signs of Suicide* (SOS) intervention [42, 43, 49], a mindfulness intervention [44], and a peer leadership training programme [51]. Only one of the effective interventions employed a more comprehensive approach consisting of a combination of training mental health nurses, cognitive behavioural therapy, and a peer leadership program [46]. Wasserman and colleagues reported that both the *Question, Persuade, and Refer* (QPR) and *ProfScreen* interventions resulted in non-significant reductions in NFSB at three-month follow-up, although significant reductions were reported at 12-month follow up [50].

Although the SOS intervention was shown to be effective in the three studies discussed above, it was also shown to be ineffective at reducing NFSB in two other studies [42, 43, 49]. Similarly, the QPR programme was also shown to be ineffective in one study [50], although a previous study had demonstrated that it was effective in reducing NFSB. The contradictory findings reported for the SOS and QPR interventions suggest that the effectiveness of these interventions might be a function of variables other than the content of the intervention, such as the way the intervention is delivered, who delivers the intervention, and the setting or context of the intervention.

#### Changing knowledge, attitudes, and stigma

Four universal interventions aimed to change knowledge and attitudes towards suicide and one intervention explicitly addressed stigma. All four interventions that targeted knowledge were found to be effective, three of which made use of the SOS intervention [42, 43, 49], and one utilised a suicide awareness curriculum [45]. These interventions were also effective at changing attitudes towards suicide when assessed immediately after the intervention, but no intervention showed sustained attitude changes in follow-up assessments [42, 43, 45, 49]. Only one universal intervention focused on stigma towards suicide, finding that psychoeducation and interpersonal exposure significantly reduced stigma of suicide at post-intervention, with change sustained at one-month follow up [48].

#### Increasing help-seeking behaviour

Three universal interventions aimed at increasing students’ ability to ask for help with suicidal thoughts and behaviours [42, 43, 52]. Two studies demonstrated that the SOS intervention did not significantly increase students’ comfort or ability to seek help and access treatment from a friend or adult [42, 43]. In contrast, the *Source Of Strengths* intervention was found to have a significant effect on positive expectations that adults would help suicidal students and increased students’ perceptions of the norms for help-seeking, but did not actually increase students’ propensity to refer distressed peers to adults [52].

### Selective interventions

Fourteen selective campus-based suicide prevention interventions were tested, all of which made use of gatekeeper training (see Table 2). Only one of these interventions was conducted on a school campus [64]. Six of these interventions focused exclusively on staff [59–64], seven focused exclusively on students [53–58, 66], while only one intervention targeted training for both staff and students [65].

**Table 2:** Main outcomes of studies on selective campus-based interventions

Authors (year of publication)	Details of the intervention	Study design	Target of the intervention	Main findings	Effect Size
**Gatekeeper interventions for students**					
Mitchell et al., 2013 [53]	Brief psychoeducation Question, Persuade, Refer (QPR) gatekeeper training	Open trial (no control group with a postintervention and 3 to 6 month follow up)	Change knowledge	Significant increase in knowledge of suicide prevention facts.	Large (*d* = 1.46)
			Increase in help-seeking behaviour	No significant increase in ability to referred anyone to on-campus mental health services.	Small (*d* = 0.12)
Pasco et al., 2012 [54]	Campus connect (didactic training and experimental exercises) gatekeeper training	Open trial (control group received a 1.5-h adapted format of Campus Connect with postintervention only)	Increase in help-seeking behaviour	Significant increase in crisis intervention skills.	Large (*d* = 1.21)^a^
Rallis et al., 2018 [55]	Brief Psychoeducation and experimental (modelled after the Campus Connect training)	Open trial (no control group and postintervention and 3 month follow up)	Change knowledge	Significant increase in declarative knowledge.	Large (*d* = 1.62)^a^
				Significant increase in perceived knowledge.	Large (*d* = 1.41)^a^
				Significant reduction in declarative knowledge at 3-month follow-up.	Large (*d* = 0.94)^a^
				Significant reduction in perceived knowledge at 3-month follow-up.	Large (*d* = 1.10)^a^
			Increase in help-seeking behaviour	Significant increase in identifying any suicidal students.	Small (*d* = 0.12)^a^
				Significant increase in making at least one referral.	Small (*d* = 0.24)^a^
Taub et al., 2013 [56]	Knowledge and crisis communications skills	Open trial (no control group and postintervention follow up)	Change knowledge	Significant increase in knowledge of suicide among new resident assistants.	Small (ηp2 = 0.16)^a^
				Significant increase in knowledge of suicide warning signs among new resident assistants.	Small (ηp2 = 0.24)^a^
				Significant increase in places to refer among new resident assistants.	Small (ηp2 = 0.30)^a^
				No significant increase in knowledge of suicide among returning resident assistants.	Small (ηp2 = 0.00)^a^
				No significant increase in suicide warning signs among returning resident assistants.	Small (ηp2 = 0.00)^a^
				No significant increase in places to refer among returning resident assistants.	Small (ηp2 = 0.00)^a^
			Increase in help-seeking behaviour	No significant prediction of crisis communication skills among new resident assistants.	Small (ηp2 = 0.00)^a^
				No significant prediction of crisis communication skills among returning resident assistants.	Small (ηp2 = 0.15)^a^
Tompkins and Witt, 2009 [57]	Brief psychoeducation Question, Persuade, Refer (QPR) gatekeeper training	Quasi-experimental non-equivalent control group design (control group option to be waitlisted or treatment as usual with postintervention and 6 month follow up)	Change knowledge	Significant increase among intervention group for self-evaluation of knowledge.	Medium (*d* = 0.51)^a^
			Increase in help-seeking behaviour	Significant increase among intervention group for perceived efficacy to refer.	Small (*d* = 0.49)^a^
Wachter Morris et al., 2015 [58]	The ALIVE @ Purdue train-the trainers program	Open trial (no control group with postintervention follow up)	Change knowledge	No significant increase in knowledge about suicide.	Medium (*d* = 0.62)^a^
				No significant increase in knowledge about potential warning signs.	Small (*d* = 0.14)^a^
				No significant increase in knowledge about places to refer.	Small (*d* = 0.00)^a^
			Increase in help-seeking behaviour	Significant increase in crisis-related communication skills.	Large (*d* = 0.95)^a^
**Gatekeeper training programmes for staff**					
Cimini et al., 2014 [59]	Gatekeeper training (tailored to group specific needs) involving didactic and experiential learning components highlighting the opportunity for behavioural rehearsal	Open trial (no control group with postintervention and 3-month follow up)	Change knowledge	Significant increase in knowledge about suicidal behaviour at postintervention.	Large (*d* = 0.78)^a^
				Significant reduction in knowledge about suicidal behaviour at follow up assessment.	Small (*d* = 0.4)^a^
			Increase in help-seeking behaviour	Significant increase in comfort level to intervene with suicidal behaviour at postintervention.	Medium (*d* = 0.74)^a^
				Significant reduction in comfort level to intervene at follow up assessment but remained significantly higher than baseline.	Medium (*d* = 0.58)^a^
Cross et al., 2010 [60]	Brief psychoeducation - QPR (Question, Persuade, Refer) gatekeeper training	Open trial (no control group with a postintervention follow up)	Change knowledge	Significant increase in knowledge about suicide at postintervention assessment.	Large (*d* = 2.28)^a^
			Increase in help-seeking behaviour	Significant increase in perceived efficacy to intervene in suicide at postintervention assessment.	Large (*d* = 2.94)^a^
Hashimoto., 2016 [61]	Gatekeeper-training based on the mental health first aid program	Single-arm follow-up study (no control group with postintervention and 1-month follow up)	Increase in help-seeking behaviour	Significant improvement in the competence of managing suicidal students and behavioural intention at postintervention.	Small (*d* = 0.46)
				Significant improvement in the competence of managing suicidal students and behavioural intention at follow-up.	Small (*d* = 0.35)
				Significant improvement in the confidence of managing suicidal students and behavioural intention at postintervention.	Medium (*d* = 0.59)
				Significant improvement in the confidence of managing suicidal students and behavioural intention at follow-up.	Small (*d* = 0.35)
Mclean et al., 2017 [62]	Adapted version of brief psychoeducation - Question, Persuade, Refer (QPR) gatekeeper training	RCT (stress and time management skills training program with a 16 weeks postintervention follow up)	Increase in help-seeking behaviour	Non-significant increase in number of interventions performed.	Small (ηp2 = 0.002)^a^
				Non-significant increase in number of times approached by a resident.	Small (ηp2 = 0.001)^a^
				Non-significant increase in number of suicidal residents reported.	Small (ηp2 = 0.005)^a^
				Non-significant increase in suicidal thought severity.	Small (ηp2 = 0.012)^a^
Shannonhouse et al., 2017 [63]	Brief Psychoeducation -Applied Suicide Intervention skills training (ASIST)	Quasi-experimental pretest–posttest design (wait-list control group with postintervention follow up only)	Change knowledge	Significant increase in knowledge about suicide across time.	Small (ηp2 = 0.28)^a^
			Change attitudes	Significant increase in participants’ attitudes about suicide across time.	Small (ηp2 = 0.32)^a^
			Increase in help-seeking behaviour	Significant increase in comfort to respond to persons-at-risk.	Small (ηp2 = 0.25)^a^
				Significant increase in competence to respond to persons-at-risk.	Small (ηp2 = 0.38)^a^
				Significant increase in confidence to respond to persons-at-risk.	Small (ηp2 = 0.14)^a^
Wyman et al., 2008 [64]	QPR (Question, Persuade, Refer) gatekeeper training versus wait-list control group	RCT (wait-list control group with postintervention and 1 year follow up)	Change knowledge	Significant increase in self-reported knowledge. No significant increase noted among staff who received a 30-min refresher training several months after initial training.	Small (*d* = 0.41)^a^
			Increase in help-seeking behaviour	Significant increase in appraisals of efficacy to perform a gatekeeper role.	Large (*d* = 1.22)^a^
				Significant increase in access to services for suicidal students.	Small (*d* = 1.07)^a^
				No significant increase in comfort in asking about suicide.	Small (*d* = 0.18)^a^
				No significant increase in referral behaviours.	Small (*d* = 0.07)^a^
				No significant increase in asking about distress.	Small (*d* = 0.27)^a^
**Gatekeeper training programmes for staff and students**					
Indelicato et al., 2011 [65]	Brief psychoeducation - QPR (Question, Persuade, Refer) gatekeeper training	Between-subjects design (no control group with 1 month and 3 month postintervention follow up)	Change knowledge	Significant increase in self-reported knowledge about suicide.	Insufficient statistics
				Significant increase in self-reported knowledge about facts on suicide prevention.	Insufficient statistics
				Significant increase in self-reported knowledge about warning signs of suicide.	Insufficient statistics
				Significant increase in self-reported knowledge about how to ask someone about suicide.	Insufficient statistics
				Significant increase in self-reported knowledge about how to persuade someone to get help.	Insufficient statistics
				Significant increase in self-reported knowledge about how to get help for someone.	Insufficient statistics
				Significant increase in self-reported knowledge about information about local resources.	Insufficient statistics
				Significant increase in self-reported knowledge about belief that asking about suicide is appropriate.	Insufficient statistics
				Significant increase in self-reported knowledge about likelihood to ask someone about thoughts of suicide if concerned for them.	Insufficient statistics
			Increase in help-seeking behaviour	Significant increase in confidence in how to respond to the situation.	Insufficient statistics
				Significant increase in comfort talking about suicide.	Insufficient statistics
				Significant increase in effectiveness of the suicide prevention intervention.	Insufficient statistics
				No significant were found regarding making a referral for help and taking the person to a mental health professional.	Insufficient statistics

^a^Effect size calculated by the authors and reported as it is reported in the original study

#### Gatekeeper training programmes for students

Six gatekeeper training interventions exclusively for students were identified. As discussed in more detail below, the outcomes of these interventions were to improve students’ knowledge about suicide (*n* = 5) and to improve students’ capacity to intervene with a peer in a suicidal crisis (*n* = 6).

Of the interventions which aimed to change students’ knowledge about suicide and where to access help, four were effective [53, 55–57] and one was ineffective [58]. The effective interventions employed *QPR* training [53, 57], brief psychoeducational and experiential training [55], and crisis communication skills training [56]. The *Alive @ Purdue Train The Trainers* program demonstrated no significant improvement in knowledge about suicide, potential warning signs, or how to refer suicidal peers to appropriate help [58].

Of the interventions that aimed to increase students’ comfort or ability to intervene with a suicidal peer, four were effective [54, 55, 57, 58] and two were ineffective [53, 56]. The *ALIVE @ Purdue Train The Trainers* program effectively improved students’ crisis-related communication skills [58]. Pasco et al. reported that a combination of didactic training and experiential exercises effectively increased students’ crisis intervention skills, although training which consisted only of didactic teaching was ineffective [54]. An adapted version of *The Campus Connect Brief Psychoeducational and Experiential Intervention* effectively enhanced students’ ability to identify suicidal peers and make at least one referral [55]. *QPR Training* [53] and *Crisis Communication Training* [56] were ineffective at improving students’ confidence and ability to intervene with a suicidal peer.

#### Gatekeeper training programmes for staff

The six staff gatekeeper training interventions focused on a range of outcomes including: changing knowledge about suicide (*n* = 4), promoting adaptive attitudes (*n* = 1), and increasing comfort or ability to intervene with a suicidal student (*n* = 6). As discussed below the effectiveness of these interventions varied widely. All four interventions which aimed to changed staff knowledge about suicide demonstrated effectiveness. Three of these interventions used *QPR* training (49,51), one employed group training using didactic and experiential learning [59], and one used the *Applied Suicide Intervention Skills Training (*ASIST) programme [63].

Only one intervention aimed at changing participant’s attitudes about suicide. Shannonhouse and colleagues found that the *ASIST* intervention produced significant and sustained improvements in participants’ attitudes towards suicide [63].

Six interventions aimed at increasing staff members’ comfort or ability to intervene with suicidal students, of which five were effective at improving staff members’ intervention skills. Of the interventions that were effective, two made use of *QPR* training [60, 64], one made use of didactic and experiential learning in groups [59], and one made use of the ASIST programme [63]. Mclean and colleagues reported that the *QPR* intervention did not significantly increase the number of times staff members intervened with a suicidal student,, number of times staff were approached by a resident student, or the number of suicidal students identified by staff [62].

#### Gatekeeper training programmes for staff and students

Only one gatekeeper training intervention targeted both staff and students [65]. This intervention consisted of brief psychoeducation using the *QPR* programme, and effectively improved participants’ confidence to respond to a suicidal crisis and comfort talking about suicide, but was ineffective at improving participants’ ability to refer a suicidal student to a mental health professional.

### Indicated interventions for high-risk students

Fifteen highly focused indicated interventions for high-risk students were identified (see Table 3), ten of which were conducted on school campuses. As discussed in more detail below, 14 of these interventions focused on reducing suicidal thoughts and behaviours and one aimed at improving high-risk students’ readiness to seek help and reduce their experience of stigma.

**Table 3:** Main outcomes of studies on indicated interventions for high-risk students

Authors (year of publication)	Details of the intervention	Study design	Target of the intervention	Main findings	Effect Size
Eggert et al., 1995 [67]	At risk high school students - assessment protocol plus 1-semester Personal Growth Class (PGC l)	Controlled before and after study (care-as-usual control group with a postintervention follow up)	Reduce suicidal behaviour	A total of 85% of the youth in Groups I reduced suicide-risk behaviours by 25%, with Group I showing a greater decline in suicide-risk behaviours than Group II.	Insufficient statistics
Eggert et al., 1995 [67]	At risk high school students - assessment protocol plus 2- semesters Personal Growth Class (PGC ll)	Controlled before and after study (care-as-usual control group with a postintervention follow up)	Reduce suicidal behaviour	A total of 65% of Group II showed reduced suicide-risk behaviours by 25%.	Insufficient statistics
Eggert et al., 2002 [68]	Counselors-CARE (C-CAST): assessment interview, counselling session, and social ‘connections’ intervention	Controlled before and after study (care-as-usual control group with a postintervention and 10 week follow up)	Reduce suicidal behaviour	Statistics were not reported for interventions and control separately. Group x Trend Interaction demonstrate that the pattern of change differed significantly between at least one of the three groups. Not clear from the stats how these differed.	Insufficient statistics
Eggert et al., 2002 [68]	CAST: combination of the C-CARE intervention (i.e. assessment interview, counselling session, and social ‘connections’ intervention) followed by a small group prevention program	Controlled before and after study (care-as-usual control group with a postintervention and 10 week follow up)	Reduce suicidal behaviour	Statistics were not reported for interventions and control separately. Group x Trend Interaction demonstrate that the pattern of change differed significantly between at least one of the three groups. Not clear from the stats how these differed.	Insufficient statistics
Fitzpatrick et al., 2005 [69]	Brief video intervention regarding problem solving and coping skills	RCT (time-matched intervention focusing on physical health issues for control group with a 1 week, 2 weeks, and 1-month postintervention follow up)	Reduce suicidal ideation	No significant difference between intervention and control group with regard to suicidal ideation at baseline.	Insufficient statistics
Fukumori et al., 2017 [70]	Three-day individual intervention program of structured writing that incorporates the emotional regulation group program and the DBT workbook	RCT (wait-list control group with a postintervention, 2 week and 1-month follow up)	Reduce suicidal ideation	No significant reduction in suicidal ideation.	Small (*d* = 0.35)^a^
Hetrick et al., 2017 [71]	Internet-based cognitive behavioural therapy (Reframe-IT)	RCT (treatment-as-usual control group with a 10 week and 22 week postintervention follow up)	Reduce suicidal ideation	No significant reduction in suicidal ideation at postintervention assessment.	Small (*d* = −0.35)
King et al., 2015 [72]	Electronic bridge mental health services (eBridge)	RCT (treatment-as-usual control group with an 8 week postintervention follow up)	Increase in help-seeking behaviour	Significant increase in readiness to intervene with own suicidal behaviour by talking to family.	Large *(d* = 2.74)^a^
				Significant increase in readiness to intervene with own suicidal behaviour by talking to a friend.	Large (*d* = 2.48)^a^
				Significant increase in readiness to intervene with own suicidal behaviour by seeing a mental health professional.	Large (*d* = 3.16)^a^
				No significant increase in readiness to seek information.	Large (*d* = 1.60)^a^
				No significant increase in readiness to seek out self-help or a support group.	Small (*d* = 0.50)^a^
				No significant increase in readiness to seek academic support services.	Small (*d* = −0.44)^a^
			Reduce stigma	Significant reduction in level of personal stigma scores at postintervention.	Large (*d* = −1.07)^a^
				Significant reduction in level of perceived public stigma at postintervention.	Medium (*d* = −0.59)^a^
Lin et al., 2019 [73]	Cognitive therapy group program	RCT (cognitive therapy control group with a 4-, 8-, 20-, and 32-week postintervention follow up)	Reduce suicidal ideation	Significant reduction in suicidal ideation at 4 weeks follow up.	Large (*d* = 5.24)
				Significant reduction in suicidal ideation at 8 weeks follow up.	Large (*d* = 4.39)
				Significant reduction in suicidal ideation at 20 weeks follow up.	Large (*d* = 3.67)
				Significant reduction in suicidal ideation at 32 weeks follow up.	Large (*d* = 3.30)
			Reduce suicidal behaviour	Significant reduction in suicide attempt 4 week follow up.	Small (*d* = 0.32)
				Significant reduction in suicide attempt at 8 weeks follow up.	Small (*d* = 0.23)
				Significant reduction in suicide attempt at 20 weeks follow up.	Small (*d* = 0.18)
				Significant reduction in suicide attempt at 32 weeks follow up.	Small (d = 0.14)
Lin et al., 2019 [73]	Dialectical behaviour therapy group program	RCT (cognitive therapy control group with a 4-, 8-, 20-, and 32-week postintervention follow up)	Reduce suicidal ideation	Significant reduction in suicidal ideation found at 4 weeks follow up.	Large (*d* = 5.24)
				Significant reduction in suicidal ideation found at 8 weeks follow up.	Large (*d* = 4.39)
				Significant reduction in suicidal ideation found at 20 weeks follow up.	Large (*d* = 3.67)
				Significant reduction in suicidal ideation found at 32 weeks follow up.	Large (*d* = 3.30)
			Reduce suicidal behaviour	Significant reduction in suicide reattempt at 4 weeks follow up.	Small (*d* = 0.32)
				Significant reduction in suicide reattempt at 8 weeks follow up.	Small (*d* = 0.23)
				Significant reduction in suicide reattempt at 20 weeks follow up.	Small (*d* = 0.18)
				Significant reduction in suicide reattempt at 32 weeks follow up.	Small (*d* = 0.14)
Pistorello et al., 2012 [74]	12-month long term Dialectical Behaviour Treatment	RCT (optimised treatment-as-usual control group with a 3 month and 18 month follow up)	Reduce suicidal behaviour	Significant reduction in suicidality (i.e., suicidal thoughts and the person’s estimation of the likelihood they would consider, attempt, and die from suicide in the future).	Medium (*d* = 0.53)^a^
Tang et al., 2009 [75]	Program of Intensive Interpersonal Psychotherapy for depressed adolescents with suicidal risk (IPT-A-IN)	RCT (treatment-as-usual control group with a postintervention follow up)	Reduce suicidal ideation	Significant reduction in suicidal ideation.	Medium (*d* = −0.78)
Thompson et al., 2000 [76]	Personal Growth Semester 1	Three-group, repeated measures design (Measure of Adolescent Potential for Suicide control group with 18 week postintervention follow up)	Reduce suicidal behaviour	Significant reduction in suicide risk behaviours.	Small (*d* = 0.12)
Thompson et al., 2000 [76]	Personal Growth Semester 2	Three-group, repeated measures design (Measure of Adolescent Potential for Suicide control group with 18 week postintervention follow up)	Reduce suicidal behaviour	Significant reduction in suicide risk behaviours.	Small (*d* = 0.21)
Xavier et al., 2019 [77]	Problem solving intervention	RCT (care-as-usual control group with 1-, 3-, and 6 month follow up	Unspecified: suicidal orientation	Significant reduction in suicidal orientation at postintervention assessment.	Large (ηp *=* 0.91)^a^
				Significant reduction in suicidal orientation at 6-months follow up assessment.	Medium (ηp *=* 0.65)^a^

^a^Effect size calculated by the authors and reported as it is reported in the original study

The interventions shown to be effective at reducing suicidal thoughts and behaviours included: the *Counsellor CARE* (C-CARE) programme and a combined C-CARE plus 12 session coping and support training intervention [68]; *Personal Growth Class* programmes [67, 76]; a combination of dialectical behaviour therapy and cognitive therapy [73]; dialectical behaviour therapy alone [74]; a problem solving intervention [77]; and intensive interpersonal psychotherapy [75]. The interventions that were ineffective at reducing suicidal thoughts and behaviours among high-risk students included: a brief intervention comparing a video on problem solving skills to a time-matched intervention on physical health issues [69]; the *Reframe-IT* internet-based cognitive behavioural therapy programme [71]; and a three-day structured writing program [70]. Finally, King and colleagues found that an electronic bridge mental health service (*eBridge*) significantly decreased personal stigma scores but not perceived public stigma among high-risk college students [72].

## Discussion

The findings of this study indicate that there is a modest (*n* = 44) but growing body of research identifying effective campus-based suicide prevention strategies for use in secondary and tertiary educational institutions. Universal, selective and indicated interventions have been tested on college and high-school campuses, making it possible for administrators to identify evidence-based multi-level suicide prevention strategies. However, the need to expand research is evident from the fact that only 17 interventions were tested in RCTs, 71.4% of studies had a sample size of less than 200 participants and 65.1% showed a moderate to high-risk of bias. Furthermore, most of the studies showed only small to moderate effect sizes and some of the findings are contradictory, with a standardized intervention shown to be effective in one setting but not another. More well-designed multi-site studies are urgently needed to expand the evidence base, especially given the high rates of suicidal behaviour in this population [1, 2]. As discussed below, there are seven important observations from our findings which have implications for establishing a research agenda in this important area of public health (see Table 4 for a summary of proposed research priorities):

**Table 4:** Research priorities to advance evidence-based suicide prevention practices in high-schools and universities

• Expand research in LMICs and diverse cultural settings. • Conduct translational research to guide the cultural adaptation and application of suicide prevention interventions that have been developed and tested in high-income settings. • Develop and test interventions not premised on an “identify-and-refer” model of suicide prevention for use in low-resource environments where there are not adequate referral networks. • Increase epidmiological research and population survailance of suicdal behaviour among adolescents and young-adults in LMICs, to advocate for making suicide prevention a priority in high-schools and univeties. • Draw on implementation science research to better understand how the implementation of interventions influences their effectiveness. • Increase the number of high quality studies that have suicide deaths as the primary outcome. • Increase the use of well-designed multi-site studies to explore contextual variables influencing implementation and outcomes. • Utilise multi-site studies, where the campus is the unit of analysis and/or a key variable for assessing outcomes. • Utilise cluster randomization trials and co-ordination of studies across a large number of sites in a range of diverse settings. • Utilise well designed randomized controlled trials and pragmatic trials to culturally adapt and test gatekeeper training in LMICs.	

Firstly, it is interesting to note the focus on gatekeeper training, which is by far the most common form of campus-based suicide prevention intervention identified in this narrative synthesis. Crucially, gatekeeper training makes use of peer-to-peer support and empowers non-mental health professionals (including teaching staff and residence staff) to intervene in a suicidal crisis, making it a potentially appealing strategy in low resource environments where mental health professionals are scarce. Gatekeeper training could be seen as being aligned with task-shifting and task-sharing approaches to scaling up mental healthcare in low- and middle-income countries [78], highlighting the importance of expanding suicide prevention research to include more RCTs of gatekeeper training in low-resource settings. However, it is noteworthy that many gatekeeper training programmes developed in high income countries aim to equip staff and students to identify suicidal individuals and refer them to appropriate mental health services, thus assuming that there is a working mental healthcare system that is able to receive and respond to suicidal patients. Likewise, many of the universal interventions identified in this narrative synthesis, aim to improve help seeking behaviours and provide information about available services, which also assumes that there are accessible, affordable, and effective mental healthcare services for students. Given that appropriate mental healthcare systems may not exist in some low-resource settings, it will be necessary to develop and test interventions that are not premised on an “identify-and-refer” model of suicide prevention.

Secondly, the current research on campus-based suicide prevention strategies comes almost exclusively from industrialized high-income countries, with a lack of studies from low resource settings. This is significant, given the research showing the importance of culturally appropriate suicide prevention strategies [3, 4]. Interventions developed and shown to be effective in one cultural setting may not be effective in a different socio-cultural context. This highlights the need to expand research on campus-based suicide prevention practices in LMICs, as well as the need for translational research to guide the cultural adaptation of suicide prevention interventions developed and tested in high-income settings. Prioritizing translational research of existing interventions in LMICs will be as important as conducting pragmatic and controlled trials of novel interventions [79]. Building on this premise, the imbalance in the availability of published prevalence and risk-or-protective factor data is a serious limitation for expanding campus-based suicide prevention in LMICs where suicide may not be considered a serious public health problem due to the lack of reliable epidemiological data. For example, a review among young people in sub-Saharan Africa reported that many countries within central Africa still do not have published data on suicidal behaviour [80]. Other systematic reviews and a meta-analysis have demonstrated similar low counts of primary studies from LMICs [81–83]. The low count of available prevalence and risk-and-protective-factor research serves as a barrier to the planning of campus-based suicide prevention in these areas.

Thirdly, the studies identified in this narrative synthesis target a range of outcomes including knowledge about suicide and where to seek help, attitudes towards suicide and stigma, non-fatal suicidal behaviour, capacity and confidence to intervene in a suicidal crisis, and willingness to seek help with suicidal thoughts. However, none of the included studies report on suicide as the outcome or target of the intervention. There seems to be an implicit unexamined assumption that changing knowledge and attitudes and reducing non-fatal suicidal behaviour will automatically lead to reductions in rates of suicide. This assumption is valid if suicidal behaviour is seen on a continuum where non-fatal and fatal suicidal behaviour are conceptualized as continuous constructs driven by the same underlying dynamics. Although widely thought of in this way, it may not be valid to conceptualize fatal and non-fatal suicidal behaviour as existing on a continuum [5, 6]. We should be cautious about automatically assuming that any of the interventions identified in this review (even those that were effective and had large effect sizes) will automatically lead to a decrease in student suicide. It is important to conduct studies on campus-based interventions that explicitly target and assess changes in the rates of suicide in order to eliminate the current bias towards interventions that only address factors which are at best indirectly linked to suicide.

Fourthly it is noteworthy that interventions, such as the SOS and the QPR programmes, were shown to be effective in one setting but ineffective in another. These findings strongly suggest that the effectiveness of suicide prevention strategies is likely to be a function of contextual variables other than the content of the intervention, such as the way the intervention is delivered, who delivers it, and the context. Such findings highlight the need for future research to draw on implementation science to better understand how the implementation of interventions influences their effectiveness [8]. This observation is of course not unique to suicide prevention studies, the lack of attention to context as a key variable affecting the outcome of interventions seems to be a major blind spot in many health intervention studies [84, 85]. A failure to attend to context and how it interacts with the content of interventions in future research, will impede the development of more sophisticated campus-based suicide prevention strategies. To this end we will need trials which seek to discover what works, for whom, under what contextual circumstances. These kinds of context-sensitive research designs will need to include multi-site studies, where the campus is the unit of analysis. It is significant to note that only one of the 44 intervention studies we identified was conducted across multiple campuses, which highlights the need for more cluster randomization trials and co-ordination of studies across a large number of sites in a range of diverse settings.

Fifthly, it is remarkable how few of the studies we identified in this study made use of information and communication technologies as a medium to deliver suicide prevention interventions. Notable exceptions include the *eBridge* [72] and the *Refrem-IT* [86] programmes, both of which showed promising results. Rapid advances in digital technologies has profound implications for suicide prevention [87] and provides opportunities for novel interventions [88, 89]. This may be particularly important given the emerging literature showing the acceptability of digital mental health interventions to adolescents and young adults [90] in a wide range of countries including India [91], the UK [92], Ireland [93], Cyprus [94], and the USA [95]. The development and testing of digital suicide prevention interventions for use in high-schools and universities could be an efficient and feasible way to scale-up campus-based suicide prevention.

Sixthly, it is significant that no studies focused explicitly on Lesbian, Gay, Bisexual, Transgender, Queer or Questioning (LGBTQ+) youth, given the growing body of evidence that LGBTQ+ youth are at greater risk for suicide than their heterosexual and gender conforming peers [96]. This highlights the need for more intervention studies focused on addressing high-risk populations, such as LGBTQ + youth.

Finally, most interventions we identified in this study targeted individual level variables (including knowledge, attitudes capacity to intervene with a suicidal student and intrapsychic drivers of suicidal behaviour), with a stark absence of eco-systemic interventions focused on socio-cultural and ecological factors. It appears that other than the handful of studies that targeted stigma, campus-based suicide prevention interventions have to-date largely ignored the potential to reduce suicide rates via systemic interventions. This is noteworthy given the role of ecological factors, such as gender-based violence and bullying [20, 21], in the aetiology of suicidal behaviour among adolescents and young adults. Focusing narrowly on individual level variables, frames suicide as a problem of the individual and fails to take a holistic and integrated systems view of the individual in context. This is not an easy problem to rectify since conducting eco-systemic interventions is expensive and requires multisite intervention studies with clustered randomization of different campuses. Furthermore, it is often challenging to test campus-wide systemic interventions because of the difficulties of trying to control for the wide range of confounding variables that could potentially shape the outcome. Nonetheless, randomized controlled trials have been successfully conducted to address eco-systemic issues in educational and community settings [22], highlighting the possibilities that exist to expand the focus and methodologies currently used in campus-based suicide prevention research.

Chief among the limitations of this narrative synthesis is the fact that we only included studies published in English. Excluding other widely spoken languages such as Chinese and Spanish, has resulted in a bias towards studies conducted in western and northern hemisphere countries.

## Conclusion

Suicides among adolescents and young adults are serious public health problems which could be ameliorated through effective suicide prevention programmes on high-school and university campuses. Identifying a solid evidence-base to guide campus-based suicide prevention efforts is an important first step towards establishing best practice. The results of this narrative synthesis highlight the need for an expansion of research in this area and the possibilities that exist to widen the range of available interventions by mounting more well-designed trials with large sample sizes, promoting research in LMICs, testing interventions where reducing the incidence of suicide is the primary outcome, expanding the methods used to include translational and intervention studies, exploring the use of digital mediums to deliver interventions, and testing eco-systemic interventions.

## Supplementary Information

**Additional file 1.**

## Data Availability

Data sharing is not applicable to this review as no datasets were generated or analyzed during the current study. All information from articles included in this narrative synthesis are presented in the figures and tables.

## Abbreviations

ASIST: Applied Suicide Intervention Skills Training
LMICS: Low-and middle-income countries
NFSB: Non-fatal suicidal behaviour
PRISMA: Preferred Reporting Items for Systematic Reviews and Meta-Analyses
RCT: Randomized Controlled Trial
ROBINS-I: Risk Of Bias In Non-randomised Studies - of Interventions
SOS: Signs of Suicide
QPR: Question, Persuade, and Refer
