# Supplementary material for: Systematic review and narrative synthesis of suicide prevention in high-schools and universities: a research agenda for evidence-based practice
Source: BMC Public Health. 2021 Jun 10;21:1116. doi: 10.1186/s12889-021-11124-w (PMC8194002; doi:10.1186/s12889-021-11124-w)
Supplement: Supplementary file 1 — Additional file 1. [file 12889_2021_11124_MOESM1_ESM.docx]

**Table S1 Search strings for suicide-related behaviour interventions among university students**

| **Database and date searched** | **Search strategy** |
| --- | --- |
| **PubMed/MEDLINE (Searched 5^th^ August 2019)** | 1. (“suicide” [Mesh] OR parasuicide* OR "self-killing" OR self-injur* OR self-mutilat* OR self-harm* OR self-immolat* OR self-poison* OR self-drowning OR self-hang* OR "deliberate overdose" OR “suicide attempt” OR suicide, attempted OR “non-suicidal behaviour” OR “non-fatal suicidal behaviour” OR suicidal ideation* OR suicidal thought* OR “suicide plan” OR self-injurious behav* OR “self-harm” OR “deliberate self-harm”)  2. ("students" OR "student" OR "students"[MeSH Terms] OR college OR “universities"[MeSH Terms] OR universities OR university OR school OR "schools"[MeSH Terms])  3. (“intervention” OR “interventions”)  4. #1 AND #2 AND #3  5. randomized controlled trial [pt]  6. controlled clinical trial [pt]  7. randomized [tiab]  8. placebo [tiab]  9. clinical trials as topic [mesh: noexp]  10. randomly [tiab]  11. trial [ti]  12. #5 OR #6 OR #7 OR #8 OR #9 OR #10 OR #11  13. #4 AND #12 |
| **Cochrane library trials (Searched 5^th^ August 2019)** | 1. (suicide* OR parasuicid* OR "self-killing" OR self-injur* OR self-mutilat* OR self-harm* OR self-immolat* OR self-poison* OR self-drowning OR self-hang* OR "deliberate overdose" OR “suicide attempt” OR suicide, attempted OR “non-suicidal behaviour” OR “non-fatal suicidal behaviour” OR suicidal ideation* OR suicidal thought* OR “suicide plan” OR self-injurious behav* OR “self-harm” OR “deliberate self-harm”)  2. (students OR student OR college OR universities OR university OR school OR schools)  3. (intervention OR interventions)  7. #1 AND #2 AND #3  8. (“randomized controlled trial” OR “controlled clinical trial” OR “randomized” OR placebo OR “clinical trials” OR randomly OR trial)  9. #7 AND #8 |
| **CINAHL Plus (EBSCO) (Searched 5^th^ August 2019)** | 1. (MH “Suicide +”) 2. (parasuicide* OR "self-killing" OR self-injur* OR self-mutilat* OR self-harm* OR self-immolat* OR self-poison* OR “self-drowning” OR self-hang* OR "deliberate overdose" OR “suicide attempt” OR suicide, attempted OR “non-suicidal behaviour” OR “non-fatal suicidal behaviour” OR suicidal ideation* OR suicidal thought* OR “suicide plan” OR self-injurious behav* OR “self-harm” OR “deliberate self-harm”) 3. S1 OR S2 4. (MH "Students+") OR (MH "Colleges and Universities+") OR (MH "Schools+") 5. (“intervention” OR “interventions”) 6. S3 AND S4 AND S5 7. (“randomized controlled trial” OR “controlled clinical trial” OR “randomized” OR placebo OR “clinical trials” OR randomly OR trial) 8. S6 AND S7 |
|  |  |
| **Table S1 Search strings for campus-based suicide prevention in high-schools and universities *(Continued)*** | |
| **Database and date searched** | **Search strategy** |
| **DARE (Searched 5^th^ August 2019)** | 1. (suicide OR parasuicide OR self-killing OR self-injury OR self-mutilation OR self-harm OR self-immolation OR self-poisoning OR self-drowning OR self-hanging OR deliberate overdose OR suicide attempt OR suicide, attempted OR non-suicidal behaviour OR non-fatal suicidal behaviour OR suicidal ideation OR suicidal thought OR suicide plan OR self-injurious behaviour OR self-harm OR deliberate self-harm)  2. (students OR student OR college OR universities OR university OR school)  3. (intervention OR interventions)  4. #1 AND #2 OR #3  5. (randomized controlled trial OR controlled clinical trial OR randomized OR placebo OR clinical trials OR randomly OR trial)  6. #4 AND #5 |
| **Africa-Wide Information (EBSCOhost) (Searched 30^th^ July 2019)** | 1. (suicid* OR parasuicid* OR "self-killing" OR self-injur* OR self-mutilat* OR self-harm* OR self-immolat* OR self-poison* OR self-drowning OR self-hang* OR "deliberate overdose" OR “suicide attempt” OR suicide, attempted OR “non-suicidal behaviour” OR “non-fatal suicidal behaviour” OR suicidal ideation* OR suicidal thought* OR “suicide plan” OR self-injurious behav* OR self-harm OR deliberate self-harm)  2. (students OR student OR college OR universities OR university OR school)  3. (intervention OR interventions)  4. S1 AND S2 OR S3  5. (“randomized controlled trial” OR “controlled clinical trial” OR “randomized” OR placebo OR “clinical trials” OR randomly OR trial)  6. S4 AND S5 |

**Table S2 Sample characteristics**

| **Authors (year of publication)** | **Site of intervention**  **Intervention target population** | **Percentage of women recruited** | **Number of participants randomised to intervention** | **Intervention period in weeks** | **Duration of each contact session in minutes** | **Number of sessions** | **Study region and econmic classification of the country where the study was conducted** |
| --- | --- | --- | --- | --- | --- | --- | --- |
| Aseltine et al., 2004 [42] | Universal School: All students (peers) | 53 and 48 (two settings) | 1027 | 2 days | - | 2 | North-America; High-income |
| Aseltine et al., 2007 [43] | Universal School: All students (peers) | 51.3; 48.4 and 57.5 (three settings) | 2039 | 2 days | - | 2 | North-America; High-income |
| Britton et al., 2014 [44] | Universal School: All students (peers) | 45.5 | 52 | 6 | 12 | 30 | North-America; High-income |
| Cimini et al., 2014 [59] | Selective University: Gatekeeper | 51 | 335 | 1 day | 90 | 1 | North-America; High-income |
| Cross et al., 2010 [60] | Selective University:  Gatekeeper | 66 | 50 | 1 day | 60 | 1 | North-America; High-income |
| Eggert et al., 1995 (PGC I) [67] | Indicated School: High-risk group | 50 | 36 | 90 class days | 55 | 90 | North-America; High-income |
|  | | | | | | | |
|  | | | | | | | |
| **Table S2 Sample characteristics *(Continued)*** | | | | | | | |
| **Authors (year of publication)** | **Site of intervention**  **Intervention target population** | **Percentage of women recruited** | **Number of participants randomised to intervention** | **Intervention period in weeks** | **Duration of each contact session in minutes** | **Number of sessions** | **Study region and econmic classification of the country where the study was conducted** |
| Eggert et al., 1995 (PGC II) [67] | Indicated School: High-risk group | 50 | 34 | 180 class days | 55 | 180 | North-America; High-income |
| Eggert et al., 2002 (C-CARE) [68] | Indicated School: High-risk group | 52 | 117 | 4 | 90 | 4 | North-America; High-income |
| Eggert et al., 2002 (CAST) [68] | Indicated School: High-risk group | 52 | 103 | 6 | 60 | 12 | North-America; High-income |
| Fitzpatrick et al., 2005 [69] | Indicated University:  Suicidal students | 55 | - | 1 day | 60 | 1 | North-America; High-income |
| Fukumori et al., 2017 [70] | Indicated University:  Suicidal students | 68 | 18 | 3 consecutive days | 25 | 3 | East Asia and pacific; High-income |
| Hashimoto et al., 2016 [61] | Selective University:  Gatekeepers | 64.8 | 76 | - | 150 | 1 | East Asia and pacific; High-income |
| Hetrick et al., 2017 [71] | Indicated School: High-risk group | 82 | 24 | 10 weeks | - | 10 | East Asia and pacific; High-income |
| Indelicato et al., 2011 [65] | Universal University: Gatekeepers | 67.7 | 917 | - | - | 1 | North-America; High-income |
| Kalafat & Elias, 1994 [45] | Universal School:  All students (peers) | 43 | 71 | 1 | 45 | 3 | North-America; High-income |
| King et al., 2015 [72] | Indicated University: High-risk group | 59 | 35 | 1 day | - | 1 | North-America; High-income |
| Lin et al., 2019 [73] | Indicated University: Suicidal students | 90.5 | 42 | 8 | 120 | 8 | East Asia and pacific; High-income |
| Lin et al., 2019 [73] | Indicated University: Suicidal students | 85 | 40 | 8 | 120 | 8 | East Asia and pacific; High-income |
| Mclean et al., 2017 [62] | Selective University: Gatekeeper | 59 | 81 | 1 day | 60 | 1 | North-America; High-income |
| Mitchell et al., 2013 [53] | Selective University:  Gatekeepers | 78 | 1644 | 1day | 90 | 1 | North-America; High-income |
| Nasution et al., 2019 [46] | Universal School: All students (peers) | 69.8 | - | - | - | - | East Asia and pacific; lower-middle-income |
| Pasco et al., 2012 [54] | Selective University:  Gatekeepers | 53.8 | 85 | - | 180 | 1 | North-America; High-income |
|  | | | | | | | |
| **Table S2 Sample characteristics *(Continued)*** | | | | | | | |
| **Authors (year of publication)** | **Site of intervention**  **Intervention target population** | **Percentage of women recruited** | **Number of participants randomised to intervention** | **Intervention period in weeks** | **Duration of each contact session in minutes** | **Number of sessions** | **Study region and econmic classification of the country where the study was conducted** |
| Pasco et al., 2012 [54] | Selective University:  Gatekeepers | 47.6 | 22 | - | 90 | 1 | North-America; High-income |
| Pistorello et al., 2012 [74] | Indicated School: Suicidal students | 77.4 | 31 | upper limit 52 | 50 | M= 34 SD=10 | North-America; High-income |
| Rallis et al., 2018 [55] | Selective University:  Gatekeepers | 65.4 | 231 | - | 60 | 1 | North-America; High-income |
| Randell et al., 2001 [47] | Universal School: All students (peers) | Total sample: 48% to 59%, no breakdown per intervention | 117 | 6 | 120 | 12 | North-America; High-income |
| Randell et al., 2001 [47] | Universal School: All students (peers) | Total sample: 48% to 59%, no breakdown per intervention | 103 | 9 | 60 | 13 | North-America; High-income |
| Rogers et al., 2018 [48] | Universal University: Undergraduate psychology students | 63.9 | Psychoeducation= 90 IP exposure= 86 | 1 day | 20 minutes | 1 | North-America; High-income |
| Shannonhouse et al., 2017 [63] | Selective University: Gatekeepers | 70 | 50 | - | 840 | 1 | North-America; High-income |
| Schilling et al., 2016 [49] | Universal School: All students (peers) | 41.7 | 729 | - | - | - | North-America; High-income |
| Tang et al., 2009 [75] | Indicated School: Suicidal students | 65.8 | 35 | 6 | 50 min face to face 30 min telephonic | 12 | East Asia and pacific;  high-income |
| Taub et al., 2013 [56] | Selective University: Gatekeepers | 50 | 300 | - | 120 | 1 | North-America; High-income |
| Thompson et al., 2000 (PGC I) [76] | Indicated School: Suicidal students | 58.3 | 36 | 18 | 55 | 90 | North-America; High-income |
| Thompson et al., 2000 (PGC II) [76] | Indicated School: Suicidal students | 62.9 | 35 | 36 | 55 | 180 | North-America; High-income |
| Tompkins and Witt, 2009 [57] | Selective University: Gatekeepers | 59 | 122 Follow up (60) |  | 60 | 1 | North-America; High-income |
| Wachter Morris et al., 2015 [58] | Selective University: Gatekeeper | 66.7 | 12 | - | - | - | North-America; High-income |
| Wasserman et al., 2015 (QPR) [50] | Universal School:  All peers | 63 | 2692 | 4 | - | - | Europe and Central Asia; High-income |
|  | | | | | | | |
| **Table S2 Sample characteristics *(Continued)*** | | | | | | | |
| **Authors (year of publication)** | **Site of intervention**  **Intervention target population** | **Percentage of women recruited** | **Number of participants randomised to intervention** | **Intervention period in weeks** | **Duration of each contact session in minutes** | **Number of sessions** | **Study region and econmic classification of the country where the study was conducted** |
| Wasserman et al., 2015 (YAM) [50] | Universal School:  All students (peers) | 60 | 2721 | 4 | 240 | - | Europe and Central Asia; High-income |
| Wasserman et al., 2015 (PofScreen) [50] | Universal School:  All students (peers) | 58 | 2764 | 4 | - | - | Europe and Central Asia; High-income |
| Wulandari et al., 2019 [51] | Universal School:  All students (peers) | 55.8 | 43 | 3 | - | 4 | East Asia and pacific; lower-middle-income |
| Wyman et al., 2008 [64] | Selective School: Gatekeepers | 81.9 | 166 | 1 | training = 960  refresher = 30 | 2 | North-America; High-income |
| Wyman et al., 2010 [52] | Universal School:  All students (peers) | 70.9; 60.1 and 76.3 across three settings | Georgia = 6888 NY = 2485 North Dakota = 293 | - | - | - | North-America; High-income |
| Xavier et al., 2019 [77] | Indicated School: Suicidal students | 60 | 50 | 5 | 120 | 5 | Latin America and the Caribbean; upper-middle-income |

**Table S3 Risk of bias summary for RCTs**

| **Authors (year of publication)** | **Bias due to the randomization process** | **Bias due to the intended interventions** | **Bias due to missing outcome data** | **Bias due to measurement of the outcome** | **Bias in selection of reported result** | **Overall bias** |
| --- | --- | --- | --- | --- | --- | --- |
| Aseltine et al., 2004 [42] | Some concerns | Low risk | Low risk | Some concerns | Some concerns | Some concerns |
| Aseltine et al., 2007 [43] | Some concerns | Low risk | Low risk | Some concerns | Some concerns | Some concerns |
| Britton et al., 2014 [44] | Low risk | Low risk | Low risk | Low risk | Low risk | Low risk |
| Fitzpatrick et al., 2005 [69] | Some concerns | Low risk | Low risk | Low risk | Low risk | Some concerns |
| Fukumori et al., 2017 [70] | High risk | Some concerns | High risk | High risk | Low risk | High risk |
| Hetrick et al., 2017 [71] | Low risk | Low risk | Low risk | Low risk | Low risk | Low risk |
| King et al., 2015 [72] | Low risk | Low risk | Low risk | Low risk | Low risk | Low risk |
| Lin et al., 2019 [73] | Low risk | Low risk | Low risk | Low risk | Low risk | Low risk |
| Mclean et al., 2017 [62] | High risk | Low risk | High risk | High risk | Low risk | High risk |
| Pistorello et al., 2012 [74] | Low risk | Low risk | Low risk | Low risk | Low risk | Low risk |
| Rogers et al., 2018 [48] | Low risk | Low risk | Low risk | Some concerns | Low risk | Some concerns |
| Schilling et al., 2016 [49] | Some concerns | Some concerns | High risk | High risk | Low risk | High risk |
| Tang et al., 2009 [75] | Some concerns | Some concerns | Low risk | Low risk | Low risk | Some concerns |
| Wasserman et al., 2015 [50] | Low risk | Low risk | Low risk | Low risk | Low risk | Low risk |
| Wyman et al., 2008 [64] | Some concerns | Low risk | Low risk | Low risk | Low risk | Some concerns |
| Wyman et al., 2010 [52] | Some concerns | Low risk | Low risk | Low risk | Low risk | Some concerns |
| Xavier et al., 2019 [77] | Low risk | Low risk | Low risk | Low risk | Low risk | Low risk |

| **Table S4 Risk of bias summary for non-RCTs** | | | | | | | | | |
| --- | --- | --- | --- | --- | --- | --- | --- | --- | --- |
| **Authors (year of publication)** | **Bias due to confounding** | **Bias in selection of participants into study** | **Bias in classification of intervention** | **Bias due to deviations from intended intervention** | **Bias due to missing data** | **Bias in measurement of outcomes** | **Bias in selection of reported result** | **Overall risk of bias** |  |
| Cimini et al., 2014 [59] | Serious risk | Low risk | Low risk | Low risk | No information | Low risk | Low risk | Moderate risk |  |
| Cross et al., 2010 [60] | Moderate risk | Low risk | Low risk | Low risk | Low risk | Low risk | Low risk | Moderate risk |  |
| Eggert et al., 1995 [67] | Low risk | Low risk | Low risk | Low risk | Low risk | Low risk | Low risk | Low risk |  |
| Eggert et al., 2002 [68] | Low risk | Low risk | Low risk | Low risk | Low risk | Low risk | Low risk | Low risk |  |
| Hashimoto., 2016 [61] | Serious risk | Moderate risk | Low risk | Low risk | Low risk | Low risk | Low risk | Moderate risk |  |
| Indelicato et al., 2011 [65] | Moderate risk | Low risk | Low risk | Low risk | Moderate risk | Low risk | Low risk | Moderate risk |  |
| Kalafat & Elias, 1994 [45] | Moderate risk | Low risk | Moderate risk | Low risk | Low risk | Low risk | Low risk | Moderate risk |  |
| Mitchell et al., 2013 [53] | Moderate risk | Low risk | Moderate risk | Moderate risk | Moderate risk | Low risk | Low risk | Moderate risk |  |
| Nasution et al., 2019 [46] | Moderate risk | Low risk | Moderate risk | Moderate risk | Moderate risk | Low risk | Low risk | Moderate risk |  |
| Pasco et al., 2012 [54] | Moderate risk | Low risk | Low risk | Low risk | Moderate risk | Moderate risk | Low risk | Moderate risk |  |
| Rallis et al., 2018 [55] | Moderate risk | Low risk | Low risk | Low risk | Low risk | Low risk | Low risk | Moderate risk |  |
| Randell et al., 2001 [47] | Moderate risk | Low risk | Low risk | Low risk | Low risk | Low risk | Low risk | Moderate risk |  |
| Shannonhouse et al., 2017 [63] | Low risk | Low risk | Low risk | Low risk | Low risk | Low risk | Low risk | Low risk |  |
| Taub et al., 2013 [56] | Moderate risk | Low risk | Low risk | Low risk | Low risk | Low risk | Low risk | Low risk |  |
| Thompson et al., 2000 [76] | Moderate risk | Low risk | Low risk | Low risk | Moderate risk | Low risk | Low risk | Moderate risk |  |
| Tompkins and Witt, 2009 [57] | Serious risk | Low risk | Low risk | Low risk | Low risk | Serious risk | Low risk | Serious risk |  |
| Wachter Morris et al., 2015 [58] | Moderate risk | Low risk | Low risk | Low risk | Low risk | Low risk | Low risk | Moderate risk |  |
| Wulandari et al., 2019 [51] | Serious risk | Low risk | Low risk | Low risk | Serious risk | Serious risk | Low risk | Serious risk |  |

**Supplementary Figure 1 Distribution of studies over time**
